# Fabrication and Characterisation of a Photo-Responsive, Injectable Nanosystem for Sustained Delivery of Macromolecules

**DOI:** 10.3390/ijms22073359

**Published:** 2021-03-25

**Authors:** Pakama Mahlumba, Pradeep Kumar, Lisa C. du Toit, Madan S. Poka, Philemon Ubanako, Yahya E. Choonara

**Affiliations:** 1 Wits Advanced Drug Delivery Platform Research Unit, Department of Pharmacy and Pharmacology, School of Therapeutic Science, Faculty of Health Sciences, University of the Witwatersrand, Johannesburg, 7 York Road, Parktown 2193, South Africa; pakama.mahlumba@students.wits.ac.za (P.M.); pradeep.kumar@wits.ac.za (P.K.); lisa.dutoit1@wits.ac.za (L.C.d.T.); philemon.ubanako@wits.ac.za (P.U.); 2 Division of Pharmaceutical Sciences, School of Pharmacy, Sefako Makgatho Health Sciences University, Pretoria 0208, South Africa; madan.poka@smu.ac.za

**Keywords:** zein, macromolecules, photo-responsive, nanospheres, sustained release, biodegradable, injectable, hydrogel

## Abstract

The demand for biodegradable sustained release carriers with minimally invasive and less frequent administration properties for therapeutic proteins and peptides has increased over the years. The purpose of achieving sustained minimally invasive and site-specific delivery of macromolecules led to the investigation of a photo-responsive delivery system. This research explored a biodegradable prolamin, zein, modified with an azo dye (DHAB) to synthesize photo-responsive azoprolamin (AZP) nanospheres loaded with Immunoglobulin G (IgG). AZP nanospheres were incorporated in a hyaluronic acid (HA) hydrogel to develop a novel injectable photo-responsive nanosystem (HA-NSP) as a potential approach for the treatment of chorio-retinal diseases such as age-related macular degeneration (AMD) and diabetic retinopathy. AZP nanospheres were prepared via coacervation technique, dispersed in HA hydrogel and characterised via infrared spectroscopy (FTIR), X-ray diffraction (XRD) and thermogravimetric analysis (TGA). Size and morphology were studied via scanning electron microscopy (SEM) and dynamic light scattering (DLS), UV spectroscopy for photo-responsiveness. Rheological properties and injectability were investigated, as well as cytotoxicity effect on HRPE cell lines. Particle size obtained was <200 nm and photo-responsiveness to UV = 365 nm by decreasing particle diameter to 94 nm was confirmed by DLS. Encapsulation efficiency of the optimised nanospheres was 85% and IgG was released over 32 days up to 60%. Injectability of HA-NSP was confirmed with maximum force 10 N required and shear-thinning behaviour observed in rheology studies. In vitro cell cytotoxicity effect of both NSPs and HA-NSP showed non-cytotoxicity with relative cell viability of ≥80%. A biocompatible, biodegradable injectable photo-responsive nanosystem for sustained release of macromolecular IgG was successfully developed.

## 1. Introduction

Proteins and peptides have been extensively studied for various therapeutic applications because they resemble existing physiological molecules, making them highly effective in vivo. However, their large molecular size, short half-life and provocation of immune response result in low absorption and adverse effects such as elevated intraocular pressure. This is a complication in clinical application such as ocular drug targeting where the structural anatomy is complex and possesses numerous tissue barriers restricting access to the posterior segment. This has led to high demand for prolonged, non-invasive therapies to reduce the frequency of administration and improve patient adherence to treatment [1,2]. Injectable delivery systems are minimally invasive drug delivery systems are capable to extend to the restricted posterior segment of the eye while maintaining minimal pain and discomfort as opposed to the highly invasive surgical therapies.

Biodegradable nanocarriers have attained popularity in numerous biomedical applications including drug delivery design. They have a potential to increase uptake of macromolecular therapeutics into the target tissue by enhancing passage through tissue barriers and protect against harsh physiological environment to achieve localized delivery and overcome limitations experienced in macromolecular drug delivery [3,4]. Nanocarriers can be designed to suit sustained and tuneable drug release through modification of polymer chemistry, obtain responsive systems by the inclusion of responsive moieties, thus aiding in prolonging the half-life of the molecules by decelerating elimination [5,6,7]. The augmented benefit of particle size and responsiveness is essential for optimization of drug delivery.

Zein is a cost-effective hydrophobic prolamin polymer extracted from maize that easily forms self-assembled spherical nanosized particles suitable as potential drug carriers. It is also biodegradable, non-toxic, biocompatible and considered generally recognised as safe (GRAS) [4,8]. An antioxidative property in zein resultant from its high aliphatic index, fatty acid content and surface hydrophobicity has been reported [9]. These properties make zein a good candidate for biodegradable nanocarriers, encapsulate and protect both hydrophobic and hydrophilic bioactives including macromolecules [10,11,12]. Zein nanospheres require surface coating to reduce aggregation encountered during formulation. Gelatin has been investigated as a surface coat for nanomaterials [13].

Externally controlled delivery systems have been largely explored for various pharmaceutical applications due to their riveting capabilities such as adjustment of the rate of response from an external source of stimulus to accomplish minimally invasive drug delivery [14,15,16]. Photo-responsive delivery systems undergo structural transformation upon irradiation with either visible light, ultraviolet light (UV) or near infrared light (NIR). Chromophores utilise a photoreaction to convert photoirradiation to a chemical signal which is then transferred to the functional part of the particle to control its properties through photoisomerization. Natural polymers can be modified into photosensitive materials by adding a chromophore [17]. 4,4′-dihydroxyazobenzene (DHAB) is an azo dye with a chromophore (N = N) that undergoes reversible photoisomerization from *trans* to *cis* when irradiated with UV light of a certain wavelength. Light is remotely controlled and that renders it non-invasive; it is also independent of chemical environmental changes experienced at different stages of disease progression and increases prospects of targeted delivery [18,19].

Hydrogel based drug delivery systems are best suitable for the purpose of flexibility and injectability. Directly incorporating drugs into hydrogels results in a shorter drug release time as a consequence of fast diffusion caused by high water content in the hydrogel [20]. Nanocarriers improve passage through tissue and control drug release into the target tissue. However, administration of drug loaded nanocarriers as solid implantable delivery systems requires invasive surgical procedures and injection is not applicable due to aggregation and sedimentation which leads to high residue in the syringe after injection and insufficient therapeutic levels at the target site. Injectable hydrogels can overcome these drawbacks as they possess the advantage of minimal invasive administration and reach for asymmetrical target sites [21]. Drug loaded solid nanoparticles can be dispersed in an injectable hydrogel for homogeneity, ease of administration and optimal therapeutic concentrations at target tissue to improve patient adherence. In addition, hydrogel-nanoparticle combinations provide diverse synergistic properties superior to their individual composites [22,23].

The aim of this study was to develop an injectable photo-responsive delivery system for sustained release of macromolecules. In this study, zein was blended with DHAB to formulate photo-responsive nanospheres encapsulated with a monoclonal antibody Immunoglobulin G (IgG) as a model protein via coacervation method. A nanosystem comprising of photo-responsive IgG loaded azoprolamin (AZP) nanospheres coated with gelatin and dispersed in genipin (GP) crosslinked hyaluronic acid hydrogel (HA) were successfully prepared and characterised through Fourier transform infrared (FTIR) spectroscopy, thermogravimetric analysis (TGA), X-ray diffraction (XRD) and scanning electron microscopy (SEM). To our knowledge, this is the first report investigating photo-responsive AZP nanospheres dispersed in HA hydrogel for sustained delivery of macromolecules.

## 2. Results and Discussion

**Figure 1:**
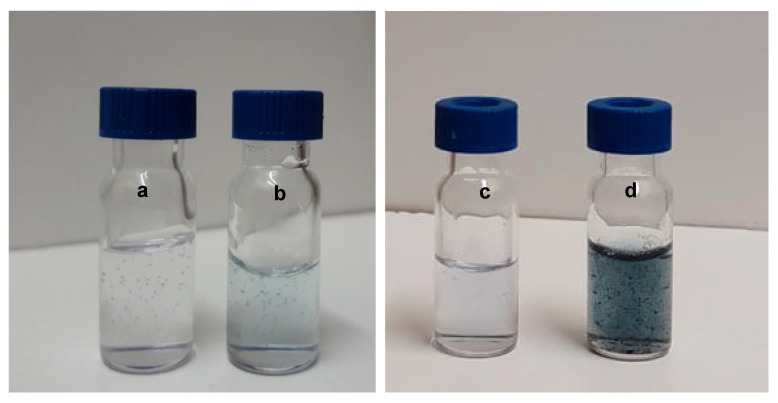
Images of the formulations (**a**) pure HA hydrogel, (**b**) HA-GP hydrogel, (**c**) pure HA hydrogel and (**d**) HA-NSP nanosystem (nanospheres + hydrogel).

IgG loaded photo-responsive AZP nanospheres were prepared by coacervation method, freeze-dried and homogenously dispersed in HA hydrogel to obtain an injectable nanosystem. Genipin was used to enhance the viscosity of the HA hydrogel in order to avoid sedimentation of the dispersed nanospheres. Genipin reacts with primary amino groups of proteins and amino acids to produce blue pigments [24]. This pigment was observed in the HA hydrogels modified with genipin. The colour variation is shown in Figure 1 where a and c are pure HA hydrogels with a clear colour, b, the HA-GP hydrogel showing a light blue colour and d, the obtained HA-NSP nanosystem displaying the dispersion of nanospheres in the hydrogel. The darker pigment in HA-NSP may be attributed to the presence of amino groups in the composition of AZP nanospheres as well as their brown colour.

### 2.1. Particle Size and Morphology

**Figure 2:**
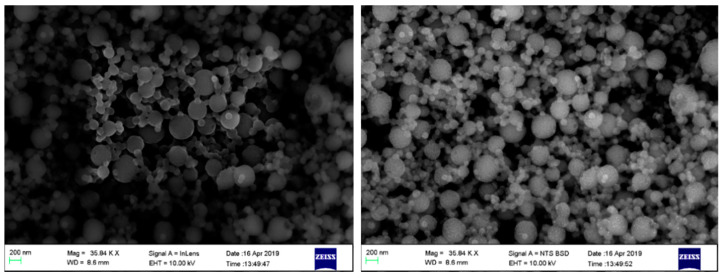
SEM micrographs of freeze-dried IgG loaded AZP nanospheres.

**Figure 3:**
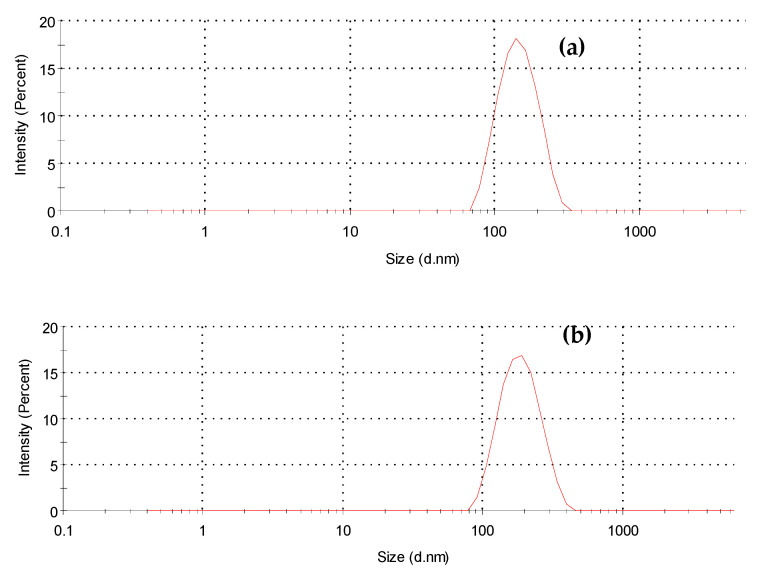
Particle size distribution for (**a**) blank (144.5 nm, PDI = 0.207) and (**b**) IgG loaded AZP nanospheres (185.5 nm, PDI = 0.190).

The size and morphology of the dried IgG loaded AZP nanospheres were examined using SEM and dynamic light scattering (DLS). The average diameter of the nanospheres ~145 nm for the blank sample and ~185 nm for IgG loaded nanospheres. The nanospheres showed a low PDI of 0.190, indicating uniformity and less aggregation in dispersion [25]. SEM micrographs in Figure 2 confirmed the spherical shape and the size of the nanospheres was <200 nm. The average diameter of the nanospheres slightly increased after the incorporation of IgG. This variation in size, displayed in Figure 3, may be attributed to the inclusion of a high molecular weight peptide [26]. A few spheres in the macro scale were observed and this large size may be due to overlapping of the nanospheres resulting from aggregation during solvent evaporation [27].

### 2.2. X-ray Diffraction

**Figure 4:**
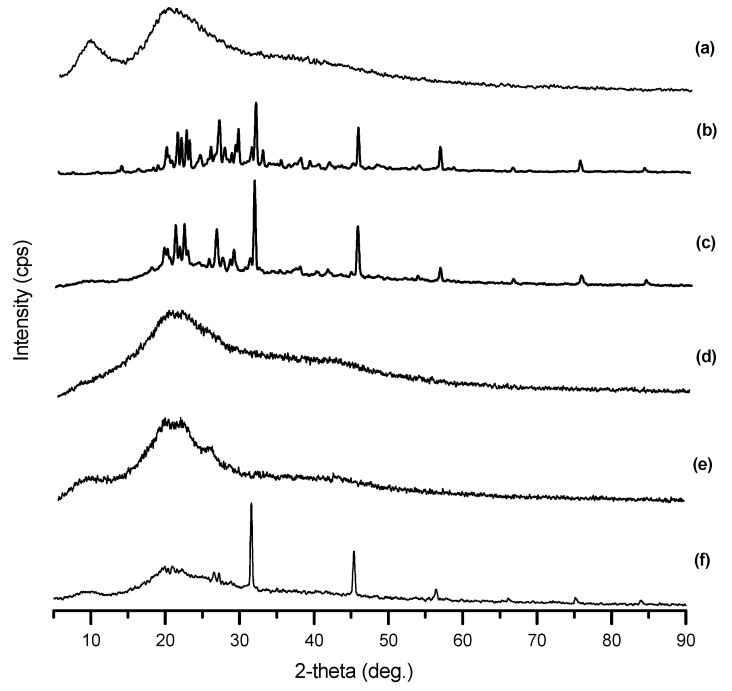
X-ray diffractograms of (**a**) zein, (**b**) AZP, (**c**) DHAB, (**d**) gelatin, (**e**) IgG and (**f**) IgG loaded AZP nanospheres.

XRD patterns for lyophilised AZP nanospheres, drug and native components are shown in Figure 4. Intense peaks were observed at 19, 22, 26, 29, 31, 45 and 56 degrees in DHAB and AZP patterns, indicative of the crystalline nature of the azo dye. On the contrary, flattened peaks were seen in zein, gelatin and IgG patterns indicating their amorphous nature. DHAB characteristic peaks, 31, 45 and 56 degrees, were seen in the nanospheres pattern substantiating the unaltered structure of DHAB and suggesting that the photoisomerization property was retained [28].

### 2.3. Fourier Transform Infrared (FTIR) Spectroscopy

**Figure 5:**
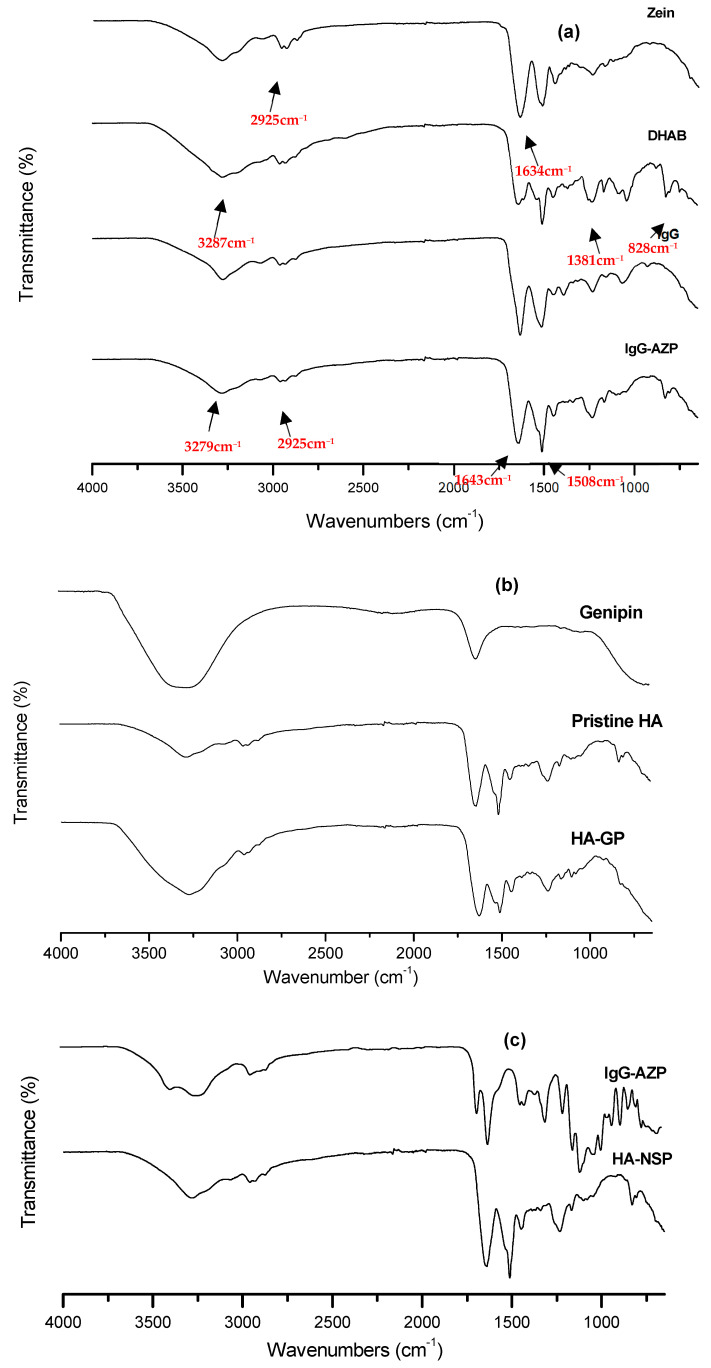
FTIR spectra of (**a**) Zein, DHAB, IgG and IgG loaded AZP nanospheres, (**b**) Genipin, Pristine HA and genipin crosslinked HA hydrogel and (**c**) IgG loaded AZP nanospheres and HA-NSP nanosystem.

FTIR was used to examine the interactions between zein, DHAB, AZP nanospheres and IgG during formulation. Spectra of the lyophilised nanospheres and native components were obtained and are presented in Figure 5a,b. Characteristic bands and peaks are seen at 828 cm^−1^ verifying the 4,4′ distribution in the ring [29], the strong absorption at 1231 cm^−1^ assigned to the aromatic C-O stretch, 1508 cm^−1^ assigned to the azo group (N = N) responsible for photo-responsiveness of the system, aromatic C-N, C = C and C-H positioned at 1381 cm^−1^,1438 cm^−1^ and 2925 cm^−1^, respectively. Amide C = O stretching, seen at 1634 cm^−1^, was accounted for by the structure of zein. The interaction between zein and DHAB results in hydrogen bonds formed from amide groups in zein and hydroxyl groups in DHAB, which is also confirmed by the right shift observed in O-H stretching bands from 3287 cm^−1^ and 3279 cm^−1^, in zein and DHAB spectra, to 3251 cm^−1^ in AZP spectrum due to the amide-hydroxyl interaction [30]. Based on these results, it was deduced that the AZP blend retained the azo group that is ascribed to the photo-responsive behaviour of the nanospheres. In Figure 5c, stretching vibrations at 3327 cm^−1^ and 3295 cm^−1^, protein amide I at (C = O) at 1635 cm^−1^ and 1643 cm^−1^, C-O stretching at 1058 cm^−1^ and 1074 cm^−1^ were seen in HA hydrogel and AZP nanospheres, respectively [31]. The presence of genipin in HA was clearly observed. There were no significant differences in the spectra of genipin modified HA hydrogel and HA-NSP; however, a slight shift in the peaks was detected in HA-NSP spectrum from 3289 cm^−1^, 1643 cm^−1^ and 1058 cm^−1^ to 3271 cm^−1^, 1627 cm^−1^ and 1050 cm^−1^. This is an indication of weak hydrogen bonding between the hydrogel and nanospheres [32].

### 2.4. Thermal Analysis

**Figure 6:**
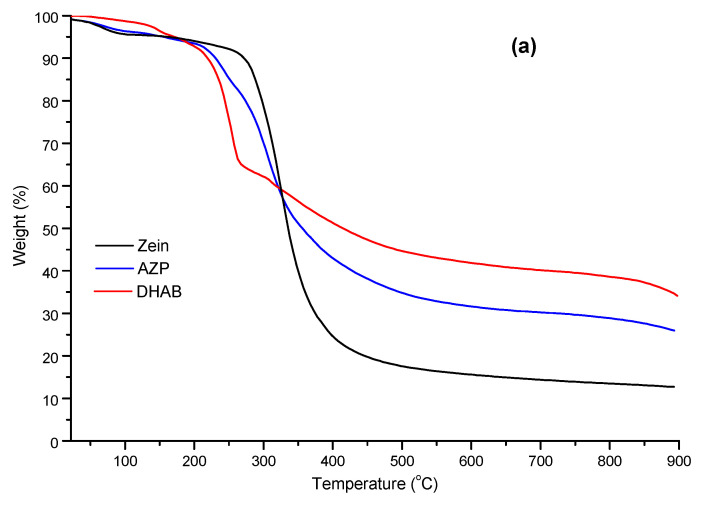

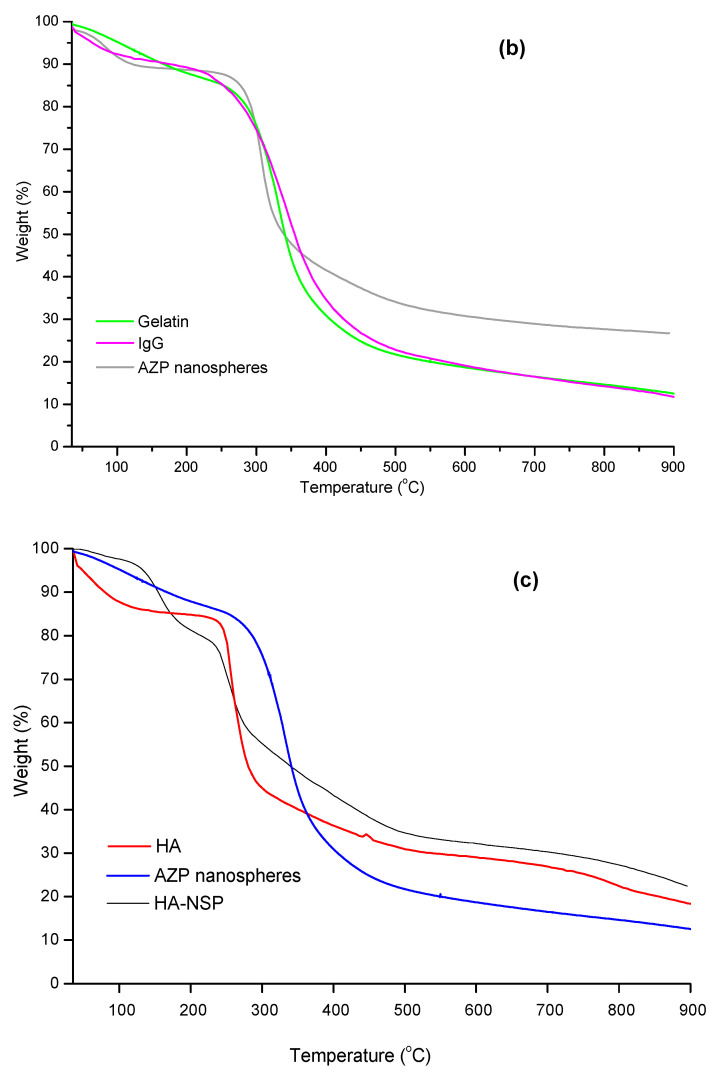
TGA thermograms of (**a**) zein, AZP and DHAB, (**b**) gelatin, IgG and IgG loaded AZP nanospheres and (**c**) HA, AZP nanospheres and HA-NSP.

Thermal stability of the lyophilised AZP nanospheres, AZP and bulk components was investigated using thermogravimetric analysis under nitrogen flow and thermograms are presented in Figure 6a and b. DHAB onset of degradation was observed at ~150 °C while other components had a lower onset of degradation temperature between 60 °C and 110 °C, indicating loss of water from the samples [26]. Zein shows thermal resistance in the initial phase but undergoes more weight loss at the end. Furthermore, DHAB had the highest stable thermal residue of 35% while zein had the lowest (13%) and the nanospheres followed a similar degradation pattern to that of AZP with the total thermal stable residue of 26%. This indicates that the presence of the crystalline DHAB increases the total stable residue and, therefore, enhances thermal resistance of both the blend and nanospheres. Thermograms displayed in Figure 6c show the degradation pattern and stability of HA-NSP and its components, AZP nanospheres and HA gel. HA-NSP underwent a two-step degradation process at 120 °C and 237 °C, whereas the components showed single degradation points with HA at 235 °C and AZP nanospheres at 280 °C. AZP nanospheres had the lowest residue (11%) and HA-NSP had the highest residues (21%), confirming its thermal stability, thus suggesting that the combination of HA gel and AZP nanospheres is more stable than the individual components.

**Figure 7:**
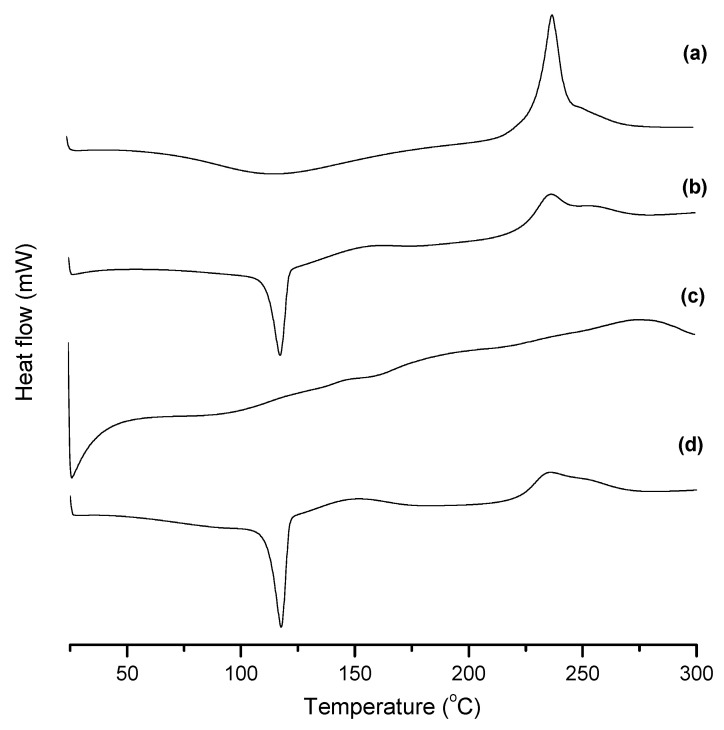
DSC thermograms of (**a**) Pristine HA, (**b**) HA gel, (**c**) NSP and (**d**) HA-NSP, measured from 25 °C to 300 °C.

Thermal behaviour of the nanospheres, HA gel and HA-NSP was analysed using differential scanning calorimetry. Samples were freeze-dried and analysed in the temperature range 25 °C–300 °C. Thermogram for pristine HA in Figure 7a showed a broad endothermic melting point at 117 °C and a sharp exothermic crystallization point at 238 °C. HA hydrogel in 7**b** displayed a sharp endothermic peak at 117 °C corresponding to its melting point and a broader exothermic peak at 238 °C suggesting that the gel is more prone to degradation compared to the native HA [33]. AZP nanospheres thermogram in **c** exhibited a broad exothermic peak at 276 °C. A distinct sharp endothermic melting point at 117 °C was observed in the thermogram for HA-NSP **d** and a broad exothermic peak at 239 °C confirming that the nanosystem’s thermal behaviour is more endothermic, therefore, the dispersed AZP nanospheres can be released from the gel without substantial energy involved in the process.

### 2.5. Determination of Photo-Responsive Property of AZP Nanospheres

**Figure 8:**
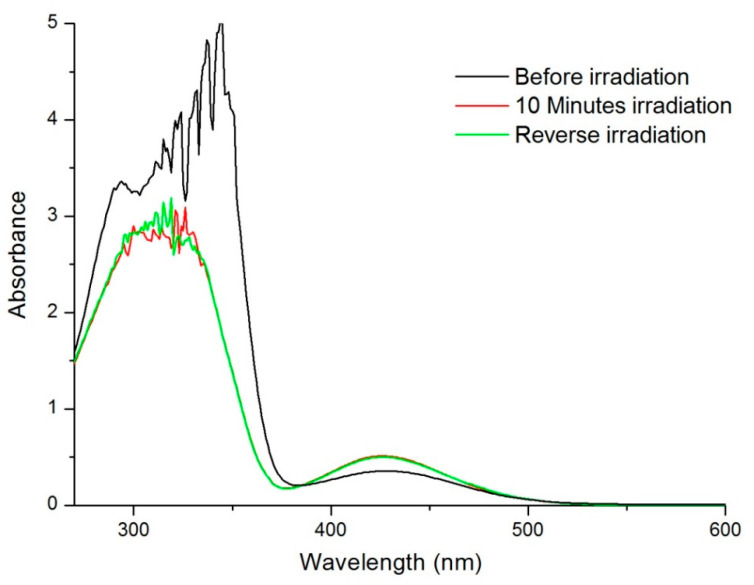
UV-vis spectra of IgG loaded AZP nanosphere dispersion post-irradiation at different time points.

To investigate photo-response, UV-Vis spectra of AZP nanosphere aqueous dispersion were obtained before and after UV irradiation and are shown in Figure 8. To incite photo-isomerisation, samples were irradiated with UV light (365 nm) to induce conversion from *trans* to the *cis* structural isomer [34]. Prior to irradiation, a maximum absorption peak was seen at 345 nm and a flat and smaller peak at 430 nm. After UV irradiation, the peak at 345 nm decreased while a slight increase in intensity in the peak at 430 nm was observed. Irradiation of the solution with white light resulted in the recovery of the peaks observed before UV irradiation. This decrease in intensity can be described, according to Ding et al. (2016), as the decrease in molar extinction coefficient caused by the reduced concentration of the *trans* isomer in the solution, concurrently, the *cis* form increases, leading to an increased intensity at 430 nm. This is accounted for by the transformation of the azo group from *trans* to *cis* isomer upon UV irradiation and recovery (*trans* to *cis*) upon exposure to white light [27,35].

**Figure 9:**
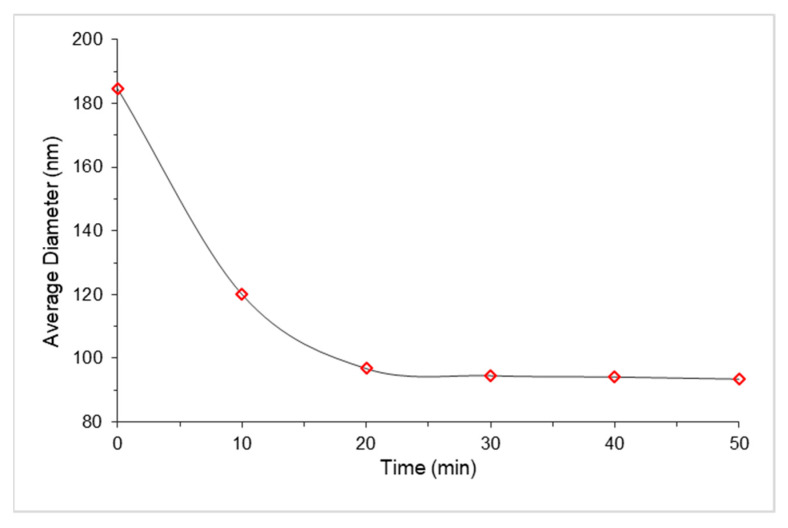
Average diameter of IgG loaded AZP nanospheres upon irradiation with UV light (365 nm) as a function of time.

Furthermore, results from dynamic light scattering are presented in Figure 9 where the change in diameter as a function of irradiation is plotted against time. The same sample was used for all time points to avoid inconsistent results. A significant decrease in the diameter of the nanosphere in the first 20 min was observed from ~185 nm to ~97 nm, followed by a constant diameter of 94 nm. These results displayed a more compact sphere after UV irradiation and this is ascribed to the photo-isomerisation of the structure from *trans* to *cis* [36].

### 2.6. Encapsulation Efficiency and In Vitro Release of IgG from AZP Nanospheres

**Figure 10:**
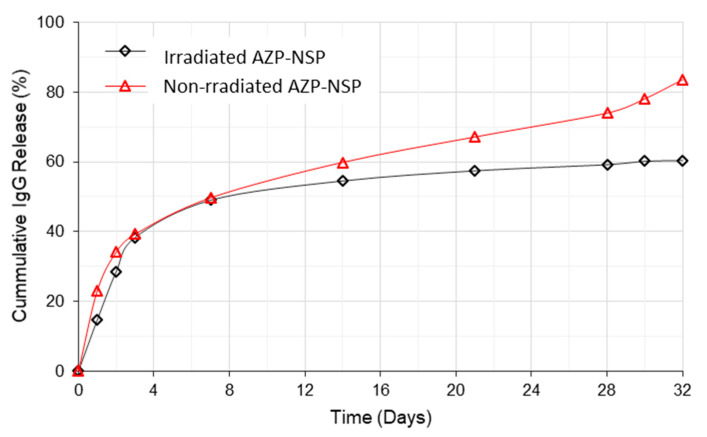
Cumulative IgG release from non-irradiated AZP nanospheres and UV irradiated AZP nanospheres post irradiation (10 min) over 32 days (*p* = 0.003).

The drug loading capacity (IgG) of the nanospheres, calculated using Equations (1) and (2), was 83%. In Figure 10, in vitro release of IgG from non-irradiated and UV irradiated AZP nanospheres is shown. In the first 24 h, 25% of IgG was released from non-irradiated nanospheres and 15% from the UV irradiated nanospheres; this initial burst release of IgG is explained as the release of free drug that is not interacted with the polymer [26]. The rest of the drug was gradually released over 4 weeks up to 60% and 84% at day 32 from irradiated and non-irradiated nanospheres, respectively. The total drug released from the UV irradiated nanospheres at day 32 was 24% less than that of non-irradiated nanospheres (*p* ≤ 0.05); this may be due to the irradiation which results in a compact nanosphere and reduction of free volume, thus decreasing rate of diffusion and prolonging the release of IgG.

**Figure 11:**
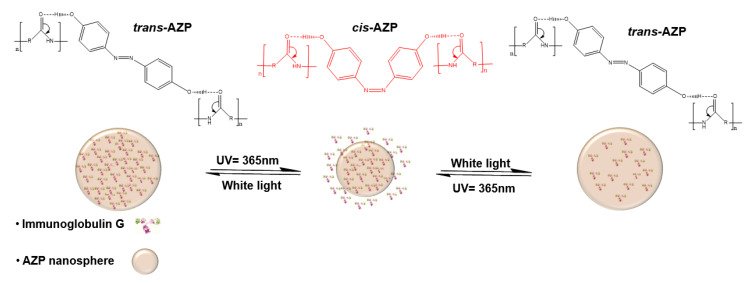
Schematic representation of the transformation from *trans* to *cis* to *tran*s and change in the size of AZP nanospheres upon UV irradiation and white light.

The proposed mechanism, previously described by Cai et al. (2014), of the reversible photo-induced IgG release, is illustrated in Figure 11, wherein, the AZP loaded nanosphere is subjected to UV light of wavelength 365 nm for a set amount of time and the *trans*-azo groups change into the *cis* form, resulting in reduced diameter of the nanosphere. The rate of diffusion decreases to allow slow release of IgG and in the reverse process where the nanospheres are irradiated with white light, azo groups reverse to *trans* form and the size of the sphere and rate of diffusion are increased [27,37].

### 2.7. Rheology

**Figure 12:**
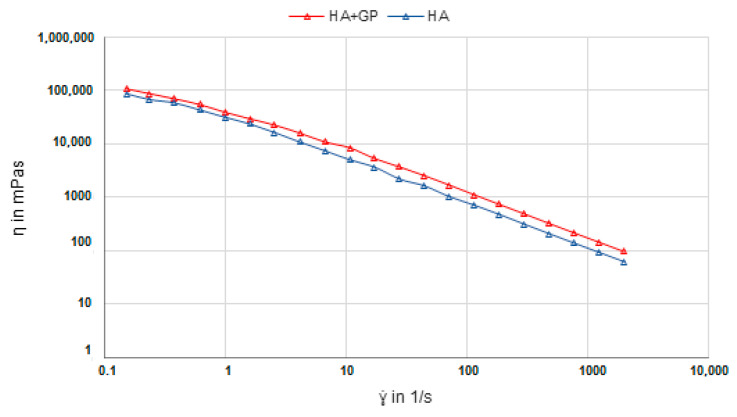
Shear viscosity measurements of pure HA hydrogel and genipin cross-linked HA hydrogel (*p* = 0.0006).

Shear viscosity measurements of the pure HA and HA-GP gels were performed to study the mechanical variation caused by the addition of genipin. In Figure 12, a slightly higher shear viscosity is observed in the HA-GP gel over the pure HA gel with a *p*-value 0.0006 substantiating the significant difference between these two hydrogels. These results confirm that adding genipin to pure HA increases viscosity of the gel. HA lacks the primary amino groups that genipin is known to react with; however, it is postulated that its highly reactive hydroxy groups are capable of forming glycosidic bonds with genipin which are stable in water [38,39].

Rheological behaviour of HA-NSP was determined through the mechanical properties which are, viscosity, elastic (G’) and viscous (G”) modulus. These are important parameters in the determination of hydrogel injectability [40]. Viscosity is a measurement of material’s resistance to deformation upon stress application and a response to shear stress variations taking place during injection of hydrogel formulations. Elastic and viscous moduli measure the elasticity or rigidity of a hydrogel and provide information about the viscoelastic response upon shear, in this case, during injection [41].

**Figure 13:**
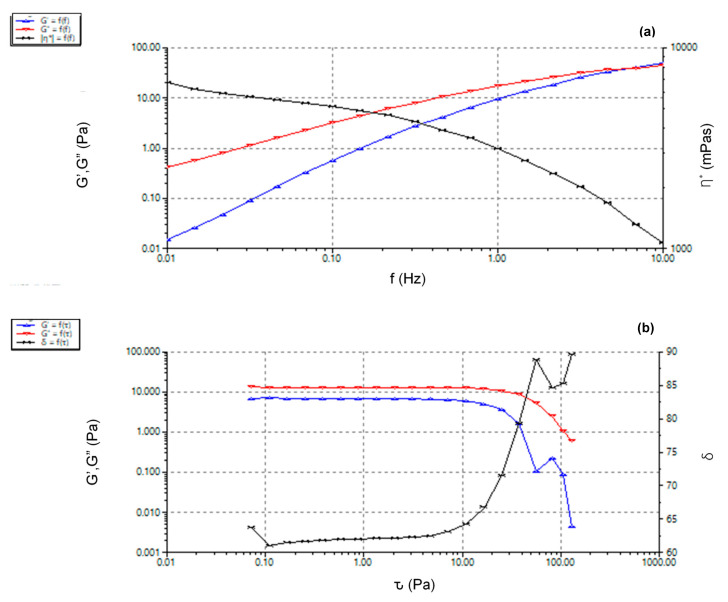
Rheology of HA-NSP (**a**) frequency sweep and (**b**)stress sweep.

A frequency sweep from 0.1 Hz to 10 Hz at 1% strain, strain sweep of up to 100 Pa and yield stress tests were conducted at 25 °C as this is the temperature of the environment in which an injection would be performed. Viscoelastic behaviour was observed in Figure 13a, wherein the loss modulus was dominant below the crossover frequency 8 Hz (G’ = G” = 75 ± 0.5 Pa) and storage modulus was dominant above this frequency. Complex viscosity decreased with the increase in frequency, indicating shear thinning behaviour of the HA-NSP nanosystem [42]. The crossover point is the transition from viscous gel character where G” > G’ to elastic behaviour, G’ > G”. Material yielding was observed from the strain sweep in Figure 13b indicated by a drop in storage and loss moduli after 47.22 Pa strain. This yield strain is the point at which material starts to flow and this is essential for ease of injection of the formulation. Viscosity decreased with increasing shear stress confirming shear thinning behaviour of HA-NSP and further indicating homogenous dispersion of the nanospheres in the gel. Shear thinning gels experience a high shear rate exerted by the walls during injection, which results in a reasonable force of injection as a consequence of the decreased viscosity [43,44].

### 2.8. Injectability

**Figure 14:**
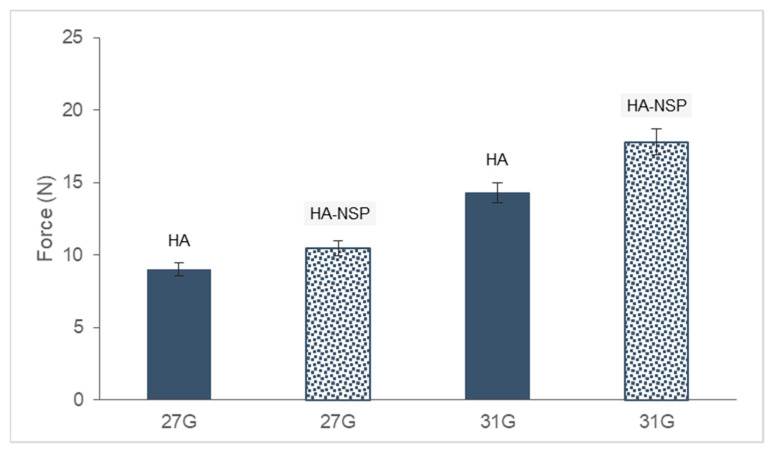
Maximum injection force for 1% HA gel and HA-NSP using two needle sizes.

Injectability of the HA gel and HA-NSP was measured as the force required to inject the hydrogel through a 1 mL syringe fitted with 27 G and 31 G needles. HA-NSP is a composition of chromophore-equipped nanospheres dispersed in HA gel. The force required for injection works on 3 parts, (i) the resistance of the syringe plunger, (ii) the kinetic energy to the contents of the syringe and (iii) forcing the liquid through the needle [45]. This process is affected by the viscosity of the sample and the size of the needle used. The maximum force required to inject hydrogels was determined via texture analysis and results are shown in Figure 14. Injectability tests were conducted on HA and HA-NSP hydrogels through 27 G and 31 G needles. HA hydrogel required 9 N and 14 N, whereas the HA-NSP hydrogel required 10 N and 18 N to inject through 27 G and 31 G needles, respectively. Two parameters that affected the force of injection are the decrease in the size of the needle and the dispersion of nanospheres in the hydrogel which resulted in increased maximum force required for injection. These results remain within two thirds of the recommended value of the maximum force for a manual injection which is 30 N; therefore, the HA-NSP is suitable for injection through various routes of administration. Furthermore, for ease of administration and minimal discomfort, 10 N is the recommended maximum force and this qualifies the 27 G needle as the best suited selection for this formulation [46].

### 2.9. Cell Cytotoxicity

**Figure 15:**
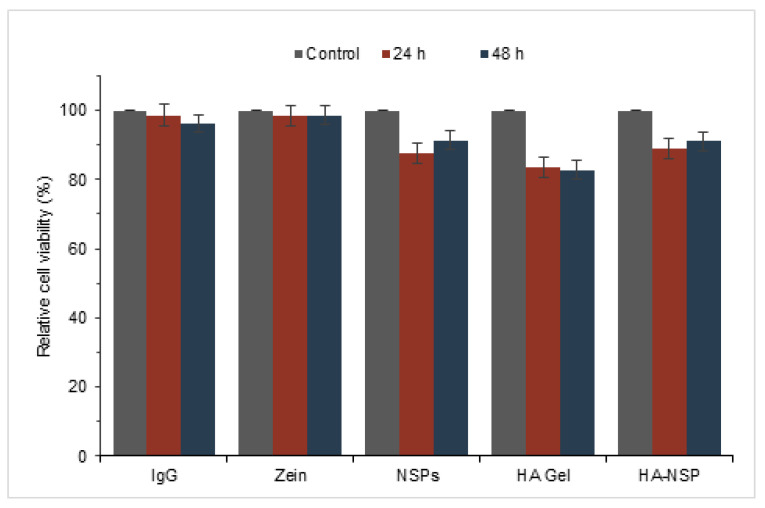
Cytotoxicity testing of HA-NSP on HRPE cells cultured for up to 48 h.

HRPE cells treated with injectable photo-responsive nanosystem (HA-NSP), NSPs, zein, HA gel and IgG solution were analysed using annexin v and dead cell assay. Results are presented Figure 15 as relative cell viability from the treated cultures of HRPE cells for up to a period of 48 h. The maximum concentrations of each component drug in the nanosystem were used as treatment. The relative viability of HRPE cells remained over 80% in all treated cultures. There was no significant change in viability from cells treated with both zein and IgG compared with untreated control cells, which confirmed the cytocompatibility of these components. Literature has attributed the cytocompatibility of zein to its degradation products that are beneficial to cell proliferation [47]. HA gel decreased cell viability by 20% over 48 h, whereas IgG loaded nanospheres and the HA-NSP decreased cell viability by 8% and 11%, respectively. Where relative cell viability is ≥70%, the material is considered non-cytotoxic [48]; therefore, these results show that the HA-NSP nanosystem is non-cytotoxic to HRPE cell lines.

**Figure 16:**
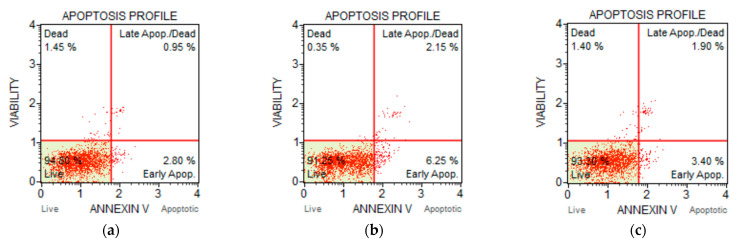

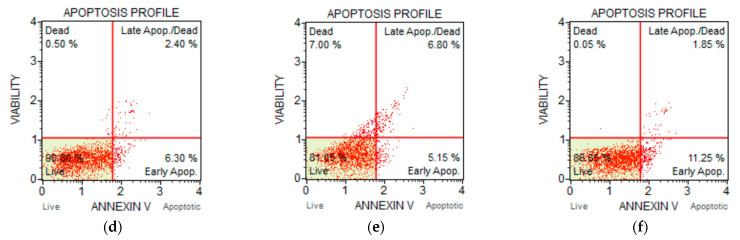
Apoptosis profiles for (**a**) untreated cells, (**b**) IgG, (**c**) zein, (**d**) NSP, (**e**) HA gel and (**f**) HA-NSP.

**Figure 17:**
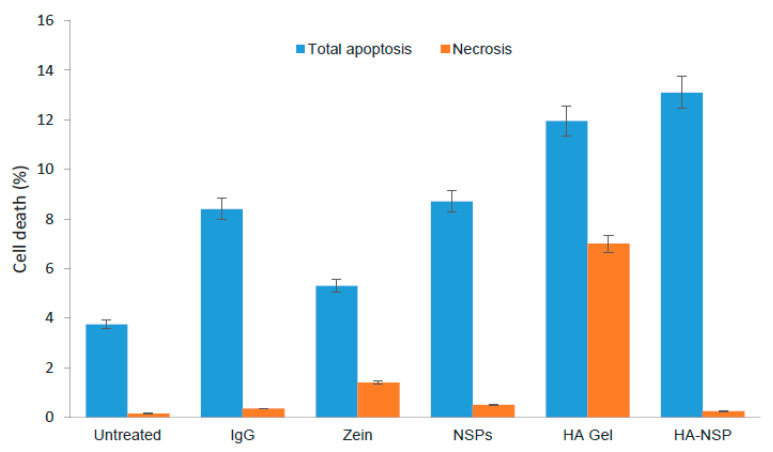
HRPE cell death presented as percentage apoptosis and necrosis (*p* = 0.004).

Annexin v binds to the externalised phosphatidylserine (PS) from asymmetrical cell membrane in apoptotic cells and the dead cell marker 7-AAD binds to necrotic cells [49]. Apoptosis eventually results in necrosis [50]. Late apoptosis is detected when the cell is positive for both PS and 7-AAD. Apoptosis profiles obtained from the study are displayed in Figure 16. Treated HRPE cell death after 48 h is presented as the percentage apoptosis and necrosis (Figure 17). These results show that the dominant pathway for cell death was apoptosis for all treatments. HA gel showed the highest percentage of necrotic cells (7%) and this may be attributed to the presence of genipin [24]; however, this percentage was 0.25% for HA-NSP confirming that cytocompatibility was improved in the composition of HA gel and AZP nanospheres.

## 3. Materials and Methods

### 3.1. Materials

Zein purified powder from maize; ethanol (EtOH) reagent grade (absolute), gelatin, 4,4′ dihydroxyazobenzene (DHAB), Immunoglobulin G from human serum, Hyaluronic acid sodium salt from streptococcus and Genipin were all purchased from Sigma-Aldrich (St. Louis, MO, USA). Sodium chloride was supplied by Merck Chemicals Pty Ltd., Germiston, South Africa.

### 3.2. Preparation of AZP Nanospheres

The azoprolamin (AZP) blend was prepared by mixing zein with DHAB in a volatile solvent under magnetic stirring overnight and dried under vacuum at 40 °C for approximately 36 h. Nanospheres were prepared via coacervation method which involves the formation of two liquid phases by partial desolvation of polymer. The solvent is evaporated by magnetic stirring at room temperature, making the formulation of the nanospheres simple and inexpensive [12,51]. The AZP blend was solubilized in 70% aqueous ethanol (0.5 mg/mL) under magnetic stirring at a speed of 300 rpm; concurrently, IgG was dissolved in 150 Mm sodium chloride solution (1 mg/mL). IgG solution was then added dropwise to the AZP solution with mild stirring for 30 min to incorporate the IgG into the nanospheres. Gelatin aqueous solution was then added with continuous stirring at room temperature to evaporate 70% ethanol. The ratio of oil to water phase was 1:4. The obtained dispersion was freeze-dried and characterized.

### 3.3. Dispersion of Nanospheres in Hyaluronic Acid Hydrogel

The nanosphere-in-hydrogel system (HA-NSP) was formulated by dispersing nanospheres in an aqueous solution and a gelling agent was added, followed by a crosslinker [22]. Genipin was used as the crosslinker and hyaluronic acid (HA) as a gelling agent. Various concentrations of HA and Genipin were explored to find the composition best suited for the nanosystem. Freeze-dried IgG loaded AZP nanospheres were dispersed in distilled water, HA was added into the dispersion stirring until completely dissolved and, finally, genipin was added to the gel to obtain the nanosphere-in-gel system.

### 3.4. Particle Size and Morphology of Nanospheres

Size distribution and polydispersity index (PDI) of the nanospheres were determined using the dynamic laser light scattering technique. Briefly, lyophilised nanospheres were dispersed in phosphate buffer saline (PBS, pH 7.4), transferred into a cuvette and subjected to laser light through the Zetasizer Nano ZS instrument (Malvern Instruments Ltd., Malvern, UK) for the measurement of particle diameter. Morphology of the nanospheres was studied via scanning electron microscopy (SEM) FEI Quanta 400 F (Hillsboro, OR, USA). Dried nanospheres were fixed onto metal stubs using double sided tape and sputter coated with a thin layer of gold under vacuum and observed under the microscope. All measurements were performed in triplicates at 25 °C, with a scattering angle of 90°.

### 3.5. X-Ray Diffraction

The physical state of the nanospheres after the formulation process and blending of zein with DHAB was assessed through X-ray diffraction (XRD) analysis. X-ray patterns of the components were obtained from X-ray diffractometer (MiniFlex 600, Rigaku, Japan), using Nickel-filtered Cu Kα radiation generated at a voltage of 40 kV and a current of 30 mA. Data were collected with a range between 5° and 90° (2θ) at room temperature, at a scanning rate of 5° min^−1^.

### 3.6. Fourier Transform Infrared Spectroscopy (FTIR)

To study chemical structures and interactions between components during formulation, infrared spectra were obtained using FT-IR spectrometer (PerkinElmer Spectrum 100). Spectra were recorded in the region from 4000 cm^−1^ to 650 cm^−1^ wavenumbers at a resolution of 4 cm^−1^. Powder samples of native components and freeze-dried nanospheres were mounted directly onto the attenuated total reflectance (ATR) crystal for analysis and to identify chemical architecture and characteristic functional groups in the formulation.

### 3.7. Thermogravimetric Analysis (TGA) and Differential Scanning Calorimetry (DSC)

Thermal decomposition of the nanosystem was observed via Thermogravimetric analysis (TGA), heated from 20 °C to 900 °C under nitrogen atmosphere at a flow rate of 20 mL/min. The thermal decomposition analyses of the samples were determined using a TGA 400 thermogravimetric analyser (PerkinElmer Inc., Waltham, MA, USA). Thermal properties of the nanosystem and its components were explored using differential scanning calorimetry (DSC) (Mettler Toledo, Schwerzernback, Switzerland). Freeze-dried samples were weighed (3–10 mg) into aluminium pans and analyzed under nitrogen atmosphere (Afrox, Germiston, Gauteng, South Africa) with 200 mL/min flow rate and the samples were then heated from 25 °C to 300 °C at the rate of 10 °C/min.

### 3.8. Photo-Responsive Property of AZP Nanospheres

The UV-vis spectra for dried nanospheres were obtained using UV spectrophotometer (Cecil CE 3021, 3000 Series, Cecil Instruments, Cambridge, UK) following a method previously explained by Ding et al. [27]. Briefly, nanospheres were dispersed in phosphate buffer saline (PBS, pH 7.4) in a 10 mm standard UV quartz cell and analysed through the UV-vis spectrophotometer. Pure PBS was used as a reference solution and samples scanned in the wavelength range of 200 nm to 800 nm for the confirmation of response to light. Additionally, the change in diameter of the nanosphere as a function of irradiation (UV = 365 nm) time was tracked by dynamic light scattering. Dried nanospheres were dispersed in PBS (pH 7.4) and exposed to UV light of 365 nm. Samples were extracted at each time point, transferred into a disposable cuvette and subjected to laser light via the Zetasizer Nano ZS instrument. All measurements were performed in triplicates.

### 3.9. In Vitro Release of IgG from AZP Nanospheres

A monoclonal antibody, Immunoglobulin G (IgG) was incorporated into the nanospheres as a model protein. Drug loading efficiency was determined by sampling a certain amount of the nanospheres and dissolving it in 70% ethanol with a probe sonicator [52]. Dried nanospheres were weighed to determine the amount recovered from formulation, particle yield was calculated using Equation (1) [11]. Particle yield, drug loading and encapsulation efficiency were calculated using Equations (2) and (3) below.

(1) $$\mathrm{Particle}\;\mathrm{yield}=\frac{\mathrm{Practical}\;\mathrm{yield}}{\mathrm{Theoretical}\;\mathrm{yield}}\;\times 100$$

(2) $$\mathrm{Drug}\;\mathrm{loading}=\left(\frac{\mathrm{Drug}\;\mathrm{mass}\;\mathrm{in}\;\mathrm{nanospheres}}{\mathrm{Mass}\;\mathrm{of}\;\mathrm{nanospheres}}\right)\times 100$$

(3) $$\mathrm{Encapsulation}\;\mathrm{efficiency}\;=\left(\frac{\mathrm{Drug}\;\mathrm{mass}\;\mathrm{in}\;\mathrm{nanospheres}}{\mathrm{Mass}\;\mathrm{of}\;\mathrm{feed}\;\mathrm{drug}}\right)\times 100$$

In vitro drug release was determined using the sample and separate method previously described by Souza, et al. [53]. Briefly, dried nanospheres were introduced into a clear cylindrical glass with PBS (pH 7.4) and incubated in a shaker bath (Orbital Shaker Incubator, LM-530, Lasec Scientific Equipment. Johannesburg, South Africa) at 20 rpm with a constant temperature of 37 ± 0.5 °C. One sample was exposed to UV light (365 nm) irradiation through a UV curing box for 10 min prior incubation in the shaker bath. A portion of the release medium was extracted and replaced with an equal volume of fresh PBS at each time point in order to maintain release conditions for the duration of the study. The amount of IgG released was measured through ultra-high performance liquid chromatography (UPLC) using acetonitrile:water:trichloroacetic acid as the mobile phase (Waters^®^ ACQUITY™LC system; Waters^®^, Milford, MA, USA) coupled with a photodiode array detector (PAD; 280 nm) and fitted with a Sunfire^TM^ C18 column with a pore size of 3.3 μm. Empower^®^ Pro Software (Waters^®^, Milford, MA, USA) was used for analysis (Figure S1).

### 3.10. Rheology and Injectability Properties

Rheological measurements were carried out on a rotational rheometer (Thermo Fisher Scientific HAAKE^TM^ MARS^TM^, Waltham, MA, USA) equipped with RheoWin software for data analysis. Samples were loaded onto the rheometer; a frequency sweep from 0.1 to 10 Hz and a strain sweep from 0.1 to 100 Pa were performed on the samples to determine storage (G’) and loss (G”) moduli. To determine the consistency and injectability of the hydrogel, the texture analyser (Stable Micro Systems TA-XT2, Surrey, UK) was used. All tests were performed in compression mode using 27 G and 31 G needles. For the test, a needle was fitted into a 1 mL syringe and placed in a holder vertically with the needle downward. A cylinder probe (P/50 R) was aligned to the plunger plate for displacement of the volume, mimicking the manual syringe injection. The compression speed was set at 1 mm/s for a distance of 20 mm. During the test, the probe compresses the syringe plunger, forcing the contents of the syringe out and the maximum force used is recorded [54,55].

### 3.11. In Vitro Cytotoxicity Testing Using H-RPE Cell Lines

Retinal pigment epithelium (RPE) is a multifunctional, nonproliferating cell monolayer located between the vascular choroid and the retina, forming the outer layer of the blood-retinal barrier. These cells function as a nutrient supplier, structural maintenance, functional integrity of the retina as well as regulating of drug transport into the inner parts of the eye [56]. RPE dysfunction is associated with the development of various retinal pathologies such as macular degeneration which may result in irreversible blindness in elderly people [57]. H-RPE cell lines were used for cytocompatibility testing on the HA-NSP 0nanosystem. Cytotoxicity effect of the HA-NSP and individual components in HRPE cell lines was assessed using the Annexin v & Dead cell assay (Millipore Corp., Billerica, MA, USA) according to the manufacturer’s protocol. Samples were analysed through the Muse^TM^ Cell Analyzer (Millipore Corp., Billerica, MA, USA) and all measurements were performed in triplicate.

## 4. Conclusions

Biodegradable photo-responsive AZP nanospheres from zein and DHAB blend were successfully developed and dispersed in HA hydrogel for injectability. The HA-NSP showed sustained release of IgG, a potential to achieve minimally invasive drug administration and slight discomfort upon injection. The photo-responsive property of the HA-NSP nanosystem is afforded by the AZP nanospheres which undergo photo-isomerization under UV light (365 nm) and their size decreased; this change in size was observed via dynamic light scattering.

Injectability of the HA-NSP nanosystem was confirmed by texture analysis in which a maximum injection force of 10 N through the 27 G needle was observed, indicative of ease of injection. These findings were supported by data from rheology, displaying shear-thinning behaviour of the HA-NSP nanosystem which is attributed to the presence of HA. Data obtained from in vitro drug release study were substantial enough to confirm the prolonged release of the macromolecular IgG in a sustained manner, up to 60% over 32 days. Results obtained from in vitro cytotoxicity testing in HRPE cells were positive as cytocompatibility was observed when cells were treated with the formulation. Though cell cytotoxicity findings suggest potential tissue compatibility of the HA-NSP nanosystem, in vivo animal studies would provide detailed data of how the delivery system behaves in an actual physiological environment.

The overall results suggest that the novel injectable HA-NSP is potentially suitable for minimally invasive site-specific delivery of any macromolecular therapeutics, such as monoclonal antibodies for age-related macular degeneration treatment. The next step includes in vivo animal testing of the HA-NSP nanosystem to examine pharmacokinetics and intraocular behaviour of the delivery system.

## Data Availability

Data is available from the researchers on request.

## Supplementary Materials

The following are available online at https://www.mdpi.com/1422-0067/22/7/3359/s1, Figure S1: Separation chromatogram of IgG.
