# Supplementary material for: Fabrication and Characterisation of a Photo-Responsive, Injectable Nanosystem for Sustained Delivery of Macromolecules
Source: Int J Mol Sci. 2021 Mar 25;22(7):3359. doi: 10.3390/ijms22073359 (PMC8037466; doi:10.3390/ijms22073359)
Supplement: Supplementary file 1 [file ijms-22-03359-s001.pdf]

Fabrication and Characterisation of a Photo-Responsive, Injectable Nanosystem for Sustained Delivery of Macromolecules

Pakama Mahlumba [1], Pradeep Kumar [1], Lisa C. du Toit [1], Madan S. Poka [2], Philemon Ubanako [1] and Yahya E. Choonara [1,*]

[1] Wits Advanced Drug Delivery Platform Research Unit, Department of Pharmacy and Pharmacology, School of Therapeutic Science, Faculty of Health Sciences, University of the Witwatersrand, Johannesburg, 7 York road, Parktown, 2193, South Africa

[2] Division of Pharmaceutical Sciences, School of Pharmacy, Sefako Makgatho Health Sciences University, Pretoria 0208, South Africa

***Corresponding Author:**
Professor Yahya E. Choonara
Tel: +27-11-717-2052
Fax: +27-11-642-4355
Email: yahya.choonara@wits.ac.za

Supplementary material

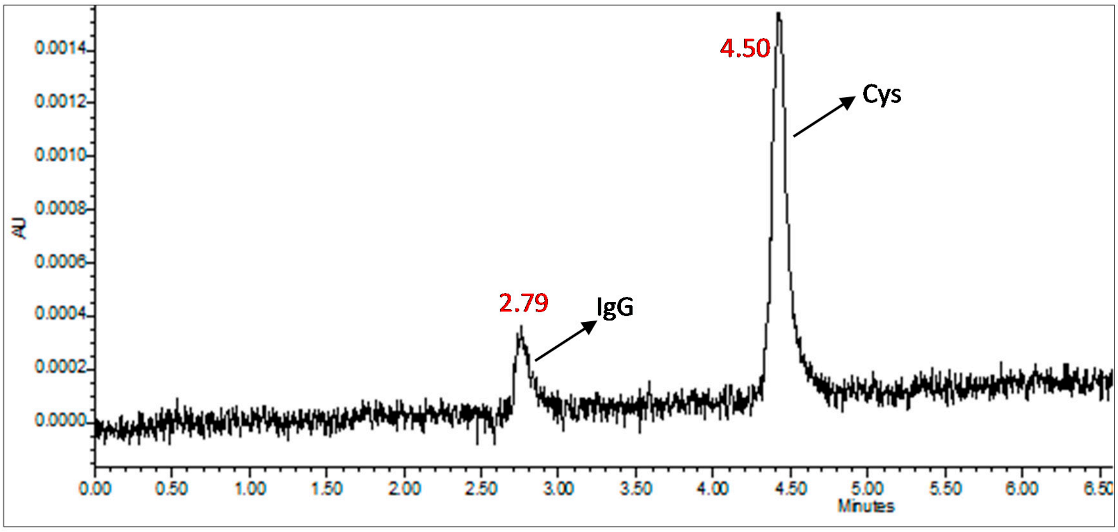

Figure S1: Separation chromatogram of IgG.
